# Why care for humanity?

**DOI:** 10.1098/rsos.231632

**Published:** 2024-04-17

**Authors:** Lukas Reinhardt, Harvey Whitehouse

**Affiliations:** ^1^ Centre for the Study of Social Cohesion, School of Anthropology and Museum Ethnography, University of Oxford, Oxford OX2 6PE, UK; ^2^ Identity and Conflict Lab, Yale University, New Haven, Connecticut, USA; ^3^ Department of Economics, University of Cologne, Cologne, Germany

**Keywords:** humanity, fusion, identity

## Abstract

Some of the most pressing challenges facing our planet—such as climate change, biodiversity loss, warfare and extreme poverty—require social cohesion and prosocial action on a global scale. How can this be achieved? Previous research suggests that identity fusion—a strong form of group cohesion motivating prosocial action—results from perceptions of shared personally transformative experiences or of common biological essence. Here, we present results from two studies with United States samples exploring each pathway to identity fusion on a global scale. Study 1 focused on globally shared motherhood experiences and found that US mothers were more fused with women around the world if they shared motherhood experiences with them, which was also reflected in money allocation behaviour. Study 2 showed that exposure to a talk about globally shared biology increased fusion with humanity at large, Americans and the extended family suggesting that fusion with humanity does not need to weaken fusion with nation or extended family. We discuss implications of our results for future research on bonding with humanity at large and for addressing collective action problems on a global scale.

## 1. Introduction

From tackling the climate crisis to preventing nuclear war, many of the world’s largest-scale collective action problems require global cohesion and prosocial action. However, human group alignments are typically parochial, prioritizing regional, national, or local interests over global ones. Is it possible to create new forms of social cohesion on a global scale, capable of transcending and overcoming parochial concerns?

It is widely known that attempts to foster large-scale identities (e.g. national identity) can foster prosocial action between group members and mitigate internal conflicts, as, for instance, the literature on nation-building demonstrates (e.g. [1–4]). However, the question of how to strengthen social cohesion on a global level is conceptually different, because there exists no human outgroup and there is surprisingly scarce evidence on this question despite the dire need for global prosocial action in the twenty-first century (for an overview, see [5]). The literature on global identity suggests that factors such as international contact [6,7], mind-body practices [8] and perceptions of equality-based respect [9] might foster global identity and prosocial action on a global scale. However, it remains a challenging task to create strong forms of global identity given that in most real-world contexts, regional, national, or local identities are stronger than global identity by far.

In this paper, we analyse the potential of ‘identity fusion’—a visceral sense of oneness with the group—to strengthen social bonds and prosocial action on a global scale. Evidence from experimental psychology and anthropology has demonstrated that identity fusion creates a strong form of social cohesion characterized by porous boundaries between personal and group identities motivating particularly strong forms of prosocial action, ranging from hypothetical willingness to sacrifice oneself to save others [10–12] to actually choosing to fight and die for the group (e.g. [13]). Other outcomes that are associated with identity fusion range from an increased willingness to trust others through to supporting other members of the group by donating blood, money or other material resources (e.g. [14,15]).

The literature on identity fusion has identified two pathways that lead to high levels of fusion: shared self-defining experiences (such as experiencing terrorist attacks, natural disasters, frontline combat, painful initiation rites or participating in extreme sports) and shared biological essence. The relationship between shared self-defining experiences and identity fusion has been studied in various contexts in correlational and quasi-experimental studies (e.g. [15]) including longitudinal designs (e.g. [16]) as well as experimental studies using tasks where study participants had to recall shared self-defining experiences (e.g. [17]). Studies that focus on the relationship between shared biological essence and identity fusion have used differences in the genetic overlap between monozygotic and dizygotic twins (e.g. [15,18]). Some studies have shown that fusion based on shared experiences can be stronger than fusion based on shared biology using priming methods (e.g. [15]) or requiring armed militia to choose between family members and fellow fighters as their primary fusion targets [13]. It has also been argued that the shared biology pathway emerges earlier in development than the shared experiences pathway [19]. Nevertheless, in adults, both pathways to fusion are associated with strong forms of pro-group action [20] and in the context of this study, our main goal is not to compare the relative strength of each pathway but to establish whether both can contribute to cohesion and prosocial action not only in local or national contexts but also on a global scale.

Social identification [21,22] also motivates pro-group behaviour. While we do not focus on or test the differences between the effects of identity fusion and the effects of identification, past studies have presented evidence that identification differs fundamentally from fusion in its developmental pathways [19], cognitive foundations [23] and downstream behavioural consequences [12]. The main conceptual difference between fusion and identification is that fusion is characterized by a synergistic relationship between personal and group identities (the one activating the other), whereas identification is characterized by a hydraulic relationship (making the one salient makes the other less so) [24]. While identity fusion and social identification in large-scale group categories may be highly correlated, even in such cases, they are established through empirically distinguishable pathways [25]. Many studies have shown that fusion produces more extreme forms of pro-group action than social identification including self-sacrifice (for an overview, see [20]). However, the main focus of this study is on whether the two hypothesized pathways to identity fusion could positively impact social bonds and prosocial behaviour on a global scale rather than on testing differences between identification and identity fusion. With regards to measurement, the pictorial scale for identity fusion we use (see [26]) is similar to the pictorial scale that is part of the 9-item ‘Identification With All Humanity Scale’ used in McFarland *et al*. [27].^1^ Both go back to the ‘Inclusion of the Other in the Self Scale’ proposed by Aron, Aron & Smollan [28]. Swann *et al*. [26] and Swann *et al*. [11] offer a more comprehensive discussion of the pros and cons of the pictorial scale as well as other measures of identification and identity fusion and the empirical relationship between both.

In the following, we present the results of two studies with United States (US) samples designed to trigger each of the two pathways to identity fusion on a global scale, respectively: perceptions of globally shared transformative experiences and species-wide shared biological essence. We see the two studies as starting points that harness the two pathways to identity fusion to strengthen social bonds on a global scale. Much more evidence is needed to properly assess the potential of these two pathways to strengthen social bonds on a global scale across diverse contexts, but the two studies we present could serve as a foundation for future endeavours.

In the first study, we focus on motherhood as a globally shared experience that transcends national, religious or political boundaries. We focus on motherhood experiences because they are highly transformative experiences [29,30] shared by mothers all over the world. Obviously, motherhood is not experienced by everybody and transformative experiences such as suffering, struggle or hope might be in one form or another truly universally shared experiences. However, the global sharedness of these latter experiences might also be perceived as abstract by many, especially since suffering, struggle or hope can take many different forms. Therefore, we have focused on globally shared motherhood experiences in this article to analyse whether transformative experiences can generate identity fusion that transcends national, religious and ethnic boundaries. We leave the analysis of other globally shared experiences that are perhaps shared by an even larger group for future research.

In the first study, we measured fusion with and money allocation (using a validated survey measure) to four groups: US mothers, US women, world’s mothers and world’s women. Our subjects are US citizens and either female and mothers or female and not mothers. Subjects who are mothers are older, less educated and politically more conservative than subjects who are not mothers, so we controlled for age, education and party preferences. Controlling for these three variables, we found that subjects who are mothers were significantly more fused with and allocated significantly more money to US mothers and world’s mothers than subjects who are not mothers, while there were no significant effects on fusion with and money allocation to US women and world’s women. However, subjects who are mothers and subjects who are not mothers probably differ with regard to many unobservable characteristics and the comparison of the two groups is no causal evidence. Therefore, we conducted within-subject comparisons. We found that subjects who are mothers were significantly more fused with and allocated significantly more money to US mothers than to US women. Subjects who are mothers were also significantly more fused with and allocated significantly more money to world’s mothers than to world’s women. These patterns did not emerge for subjects who are not mothers. Comparing these differences for subjects who are mothers and subjects who are not mothers (difference in difference) showed that there were significant ‘shared motherhood’ bonuses on fusion and money allocations both in the US context (comparing fusion with and money allocations to US mothers and US women) and in the global context (comparing fusion with and money allocations to world’s mothers and world’s women) that cannot be explained by differences in age, education or party preferences.

In the second study, we used another US sample and exposed participants in a between-subjects design to a talk about globally shared biology that made the point that we are all part of a global family through descent from a common human ancestor. We found that exposure to the talk not only significantly increased fusion with humanity but also with fellow Americans and the extended family. These results imply that fusion with humanity at large need not weaken fusion with more parochial groups such as nation or family. We did not find effects on fusion with the immediate family where shared biology is already highly salient.

We did not observe a significant ingroup bias in a validated money allocation task where subjects had to split money between a fellow American and a person from anywhere in the world. Thus, there was no room for the treatment to reduce it substantially and the treatment effect on this allocation decision was indeed not significant. However, we demonstrated that fusion mattered for money allocation behaviour by showing that fusion with humanity strongly and significantly increased the share that was allocated to the person from anywhere in the world if we controlled for fusion with Americans. The effect of fusion with Americans on the share that is allocated to the American had a similar size and was also significant if we controlled for fusion with humanity. Since the treatment increased fusion with humanity and fusion with Americans, this result suggests that higher fusion levels for both sides balance out. We found similar effects for a second money allocation task where subjects had to split money between a random American and an extended family member.

Moreover, we found that the treatment significantly increased fusion with the political outgroup (Democrats versus Republicans) and made subjects allocate money significantly more equally between Democrats and Republicans. Thus, notions of shared biology might help to address not only global problems but also to mitigate political conflicts within countries.

## 2. Study 1: design and hypotheses

In our first study, we tested whether subjects were more fused with and allocated more money to others with whom they shared a transformative experience both in a national context but also in a global context. We focused on motherhood as a shared experience because it is a personally transformative experience that is shared by mothers from all countries, religions and political factions, transcending most other forms of group alignment. It has been shown previously that childbirth experiences can create social bonds between mothers [29]. Here, we present evidence that shared experiences of motherhood can fuse mothers not only from the same neighbourhood, ethnic group or country but also globally, across national borders.

We conducted our online study on Prolific where we ordered a sample of 1000 subjects. We excluded subjects who failed the attention check, did not identify as females or stated that they were mothers but had no children. We ended up with a final sample size of 933 consisting of 481 mothers and 452 non-mothers. All subjects in the final sample were female and US citizens. The average age for mothers was 47 and 35 for non-mothers; 51% of mothers and 69% of non-mothers had a university degree. Among mothers, 47% supported the Democratic party and 26% supported the Republican party, whereas among non-mothers, 61% supported the Democratic party and 13% supported the Republican party. We paid £0.42 for subjects in the first study, resulting in an average hourly rate of £9.05 which corresponded to $11.15 on the day of data collection (31 January 2023).

The procedure of the study was as follows: first, we elicited demographics. Then, we asked subjects who are mothers to state how transformative they perceived motherhood. Non-mothers did not see this question. Then, we measured fusion of all subjects with four groups in random order: all US mothers, all US women, all the world’s mothers and all the world’s women. We used a pictorial measure for identity fusion introduced by Swann *et al.* [26]. The pictorial measure consists of five pictures. Each picture includes two circles that symbolize the self and a target group and overlap to various degrees. The subjects were asked to select the picture that best described their relationship with the target group. The pair of circles with the least overlap (weakest fusion) was coded into a value of 1 and the pair of circles with the strongest overlap (highest fusion) was coded into a value of 5. A substantial literature presents evidence that the pictorial measure of fusion predicts pro-group behaviour and especially extreme actions for the sake of the group at large personal costs [10,26].

Finally, we measured behaviour in four validated hypothetical money allocations where subjects were asked to split a hypothetical amount of $100 between two persons in each allocation. In each allocation decision, one person was a randomly selected American and the other was a member of the above-mentioned groups. These hypothetical money allocations have been validated [31,32], i.e. it was shown that hypothetical allocation decisions predict incentivized allocation decisions as accurately as a second incentivized measurement a week after the initial measurement. Therefore, our hypothetical allocation decisions are meaningful measures of how subjects weigh allocations to different groups.

We pre-registered the following hypotheses:^2^ ^,^ ^3^

– **hypothesis 1a (H1a):** all subjects are more strongly fused with US mothers than with world’s mothers; and
– **hypothesis 1b (H1b):** all subjects are more strongly fused with US women than with world’s women.

H1a and H1b hypothesize that subjects are more fused with their fellow citizens. While we are primarily interested in shared motherhood experiences, differences regarding the nationality of the target groups might provide a first plausibility check and serve as a benchmark.

– **hypothesis 2a (H2a):** mothers are more strongly fused with US mothers than with US women; and
– **hypothesis 2b (H2b):** mothers are more strongly fused with mothers of the world than women of the world.

H2a and H2b hypothesize that mothers are more strongly fused with other mothers in the US and globally.

– **hypothesis 2c (H2c):** relative fusion with US mothers versus US women (i.e. the difference) is higher for mothers with high perceived transformativeness levels; and
– **hypothesis 2d (H2d):** relative fusion with world’s mothers versus world’s women (i.e. the difference) is higher for mothers with high perceived transformativeness levels.

H2c and H2d hypothesize that the perceived transformativeness of the shared experience is associated with higher relative fusion with others who shared that experience which is in line with identity fusion theory.

– **hypothesis 3a (H3a):** subjects who are not mothers are not more strongly fused with US mothers than with US women; and
– **hypothesis 3b (H3b):** subjects who are not mothers are not more strongly fused with world’s mothers than with world’s women.

H3a and H3b hypothesize that subjects who are not mothers are not more fused with mothers than with women in general both in the US and globally. Comparing the fusion levels of mothers and non-mothers does not generate causal evidence. However, H2a–H3b are consistent with the causal claim that motherhood increases fusion with other mothers, both in the national and global context.

We pre-registered that we use difference in means tests to test our hypotheses and for H2c and H2d Ordinary Least Squares (OLS) regressions. We have not pre-registered and we did not perform multiple comparison corrections. We also pre-registered that we will test equivalent hypotheses for money allocation decisions.

## 3. Study 1: results

### 3.1. Comparisons between subjects who are mothers and subjects who are not mothers

**Figure 1.**
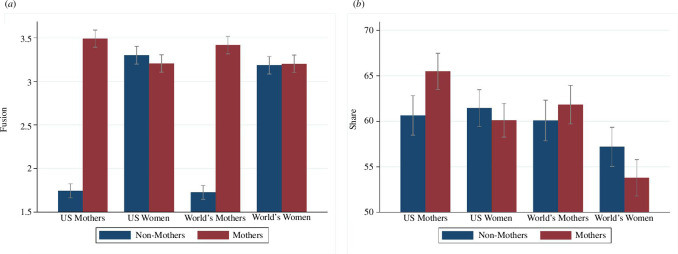
(*a*) Fusion and (*b*) money allocations. (*a*) captures fusion levels with the four target groups for subjects who are mothers (red bars) and subjects who are not mothers (blue bars). (*b*) captures money allocations to the four target groups for subjects who are mothers (red bars) and subjects who are not mothers (blue bars). Confidence intervals have confidence levels of 0.95.

Fusion levels and money allocation behaviour of subjects who are mothers and subjects who are not mothers to the four target groups are presented in figure 1. We started with a direct comparison between subjects who are mothers and subjects who are not mothers. Subjects who are mothers were more strongly fused with US mothers (OLS with robust s.e., *b* = 1.74, *p* < 0.001; table 1*a*, column 1) than subjects who are not mothers. Subjects who are mothers were also more strongly fused with world’s mothers (OLS with robust s.e., *b* = 1.69, *p* < 0.001; table 1*a*, column 5) than subjects who are not mothers. There are no significant differences for fusion with US women and fusion with world’s women. Controlling for age, education and party preferences did not change these results.

**Table 1.** Comparing subjects who are mothers and subjects who are not mothers. (*p < 0*.*1; **p < 0*.*05; ***p < 0*.*01. *Notes:* Controls include age, education, and party preferences. ‘mother’ is a dummy variable that takes the value of 1 if the subject is a mother. The outcome variable is fusion with the respective target group in (*a*) and the share that is allocated to the respective target group in (*b*). *p*-values are in parentheses. All columns present results from OLS regressions with robust s.e.).

	US mothers		US women		world’s mothers		world’s women	
	(1)	(2)	(3)	(4)	(5)	(6)	(7)	(8)
(*a*) fusion								
mother	1.747***	1.720***	−0.0951	−0.0567	1.692***	1.650***	0.0158	0.0176
	(0.000)	(0.000)	(0.188)	(0.494)	(0.000)	(0.000)	(0.827)	(0.832)
cons	1.743***	1.729***	3.301***	3.437***	1.726***	1.621***	3.186***	3.195***
	(0.000)	(0.000)	(0.000)	(0.000)	(0.000)	(0.000)	(0.000)	(0.000)
controls	no	yes	no	yes	no	yes	no	yes
*n*	933	933	933	933	933	933	933	933
(*b*) money allocations								
mother	4.838***	9.675***	−1.333	0.745	1.750	8.907***	−3.406**	2.871*
	(0.001)	(0.000)	(0.341)	(0.636)	(0.265)	(0.000)	(0.023)	(0.085)
cons	60.65***	73.07***	61.45***	65.63***	60.08***	75.90***	57.19***	69.86***
	(0.000)	(0.000)	(0.000)	(0.000)	(0.000)	(0.000)	(0.000)	(0.000)
controls	no	yes	no	yes	no	yes	no	yes
*n*	933	933	933	933	933	933	933	933

Subjects who are mothers allocated $4.84 more to US mothers than subjects who are not mothers (OLS with robust s.e., *p* = 0.001; table 1*b*, column 1). There are no significant effects regarding allocations to US women and world’s mothers and subjects who are not mothers allocated $3.41 more to world’s women than subjects who are mothers (*p* = 0.023; table 1*b*, column 7). However, subjects who are mothers were older, more conservative and less educated than subjects who are not mothers and money allocation results changed when we controlled for age, education and party preferences. In this case, there are positive effects of the motherhood status of the subject on allocations to US mothers (*b* = $9.68, *p* < 0.001; table 1*b*, column 2) and world’s mothers (*b* = $8.91, *p* < 0.001; table 1*b*, column 6), while there are no significant effects on money allocations to US women and world’s women. Thus, if we control for age, education and party preferences, the money allocation results show the same patterns as the fusion results.^4^

### 3.2. Within-subject comparisons

Subjects who are mothers and subjects who are not mothers probably differed in a lot of unobservable characteristics that affect fusion and money allocation behaviour. Hence, the comparison between both groups is not causal evidence regarding the effect of motherhood experiences. Therefore, in the following, we test our pre-registered hypotheses that focus on within-subject comparisons.

We found significant effects that support all our hypotheses concerning fusion levels. First, we show that fusion was affected by nationality. Subjects were more fused with US mothers than with world’s mothers ( $M_{FusionUSMothers}$ = 2.64; $M_{FusionWorldsMothers}$ = 2.60; *p* = 0.019; H1a)^5^ and more fused with US women than with world’s women ( $M_{FusionUSWomen}$ = 3.25; $M_{FusionWorldsWomen}$ = 3.19; *p* = 0.012; H1b).

Next, we focus on subjects who are mothers. Subjects who are mothers were more fused with fellow mothers than with women in general both in the national context ( $M_{FusionUSMothers}$ = 3.49; $M_{FusionUSWomen}$ = 3.21; *p* < 0.001; H2a) and global context ( $M_{FusionWorldsMothers}$ = 3.42; $M_{FusionWorldsWomen}$ = 3.20; *p* < 0.001; H2b).

For subjects who are mothers, the difference between fusion levels with mothers and fusion levels with women in general increased in the perceived transformativeness of motherhood both in the national context (OLS with robust s.e., *p* < 0.001; see the electronic supplementary material, table S5; H2c) and global context (OLS with robust s.e., *p* < 0.001; see the electronic supplementary material, table S6; H2d). The fact that the difference between fusion with mothers and women in general increased in transformativeness is consistent with the theoretical idea that the effect of sharing experiences is larger, the more transformative the shared experience is.

Subjects who are not mothers were more fused with women in general than with mothers both in the national context ( $M_{FusionUSMothers}$ = 1.74; $M_{FusionUSWomen}$ = 3.30; *p* < 0.001; H3a) and global context ( $M_{FusionWorldsMothers}$ = 1.73; $M_{FusionWorldsWomen}$ = 3.19; *p* < 0.001; H3b). The absolute difference between fusion with mothers and fusion with women is larger for subjects who are not mothers. This result plausibly fits with theoretical expectations. Subjects who are mothers might perceive being a woman and being a mother as well as the transformative experiences that go along with being both as essential aspects of their personal self-concept that are shared with other women and mothers and thus result in relatively high levels of fusion with mothers and with women. However, subjects who are not mothers might perceive being a woman as a central aspect of their personal self-concept but might not perceive being a mother as a central aspect of their personal self-concept and have not undergone many of the transformative experiences that go along with motherhood. Therefore, it is theoretically plausible that subjects who are not mothers have relatively high fusion levels with women but much lower fusion levels with mothers. The facts that mothers were more fused with mothers and non-mothers were more fused with women in general are consistent with the idea that the shared transformative experience of motherhood creates fusion both nationally and across national borders.

Fusion and money allocations correlated significantly for all four target groups: US mothers, US women, world’s mothers and world’s women (all *p-*values < 0.004; electronic supplementary material, table S18). However, while there were significant effects that support H1a–H2c for money allocations as the outcome, there were no significant effects for H2d and H3a for money allocations and the effect for H3b goes in the different direction.

Subjects allocated more to US mothers than to world’s mothers ( $M_{ShareUSMothers}$ = $63.14; $M_{ShareWorldsMothers}$ = $60.98; *p* < 0.001; H1a) and more to US women than to world’s women ( $M_{ShareUSWomen}$ = $60.76; $M_{ShareWorldsWomen}$ = $55.43; *p* < 0.001; H1b).

Subjects who are mothers allocated more to US mothers than to US women ( $M_{ShareUSMothers}$ = $65.49; $M_{ShareUSWomen}$ = $60.11; *p* < 0.001; H2a) and more to world’s mothers than to world’s women ( $M_{ShareWorldsMothers}$ = $61.83; $M_{ShareWorldsWomen}$ = $53.78; *p* < 0.001; H2b). These effects go in the same direction as the effects on fusion. For subjects who are mothers, the difference between allocations to mothers and allocations to non-mothers increased in the perceived transformativeness of motherhood in the national context (OLS with robust s.e., *p* = 0.044; electronic supplementary material, table S14; H2c). In the global context, the effect is not significant (OLS with robust s.e., *p* = 0.413; electronic supplementary material, table S15; H2d).

For subjects who are not mothers, there was no significant difference between allocations to US women and US mothers ( $M_{ShareUSMothers}$ = $60.65; $M_{ShareUSWomen}$ = $61.45; *p* = 0.434; H3a). Moreover, subjects who are not mothers allocated more to world’s mothers than to world’s women ( $M_{ShareWorldsMothers}$ = $60.08; $M_{ShareWorldsWomen}$ = $57.19; *p* < 0.001; H3b). The latter effect does not go in the same direction as the respective effect on fusion, which is an unexpected result. The fact that the term ‘world’s mother’ might cue association with charity and potential concerns about child wellbeing might have contributed to this result. In addition to these potential motives, there could also be effects due to the mere salience of children in an international context that affects prosociality [33]. However, regardless of such a ‘world’s mothers’ fixed effect that increased allocations to world’s mothers for all subjects, it is still the case that the difference between what was allocated to world’s mothers and what was allocated to world’s women is larger for subjects who are mothers themselves as the following difference in difference results show.

Table 2 includes difference in difference results comparing within-subject differences of subjects who are mothers and subjects who are not mothers. The dependent variable in column 1 is the difference between fusion with US mothers and fusion with US women where high values indicate that the subject was more strongly fused with US mothers than with US women. Column 1 shows that this difference is significantly higher for subjects who are mothers than for subjects who are not mothers. Adding controls in column 2 does not meaningfully change the effect size or the *p*-value suggesting that the difference in difference results cannot be explained by age, education or party preferences. Columns 3 and 4 include equivalent regressions for the global context while columns 4–8 include equivalent regressions for money allocations. Coefficients are stable after adding controls in all four cases and even slightly increase in the money allocation regressions. The results show that mothers (as compared to non-mothers) were more fused with other mothers than with women in general and allocated more money to other mothers than to women in general in the US context and global context. Thus, we see highly significant ‘shared motherhood’ bonuses on fusion and money allocations in the US and global context which cannot be explained by differences in age, education or party preferences.

**Table 2.** The difference in difference results. (*p < 0*.*1; **p < 0.05.; ***p < 0*.*01. *Notes:* In columns 1–4, the dependent variable is the difference between fusion with mothers and fusion with women. In columns 5–8, the dependent variable is the difference between allocation to mothers and allocation to women. Columns 1, 2, 5, and 6 capture the US context while columns 3, 4, 7, and 8 capture the global context. Controls include age, education, and party preferences. ‘mother’ is a dummy that takes the value of 1 if the subject is a mother and 0 otherwise. *p*-values are in parentheses. All columns present results from OLS regressions with robust s.e.).

	fusion US		fusion world		allocation US		allocation world	
	(1)	(2)	(3)	(4)	(5)	(6)	(7)	(8)
mother	1.842***	1.776***	1.676***	1.633***	6.171***	8.929***	5.156***	6.035***
	(0.000)	(0.000)	(0.000)	(0.000)	(0.000)	(0.000)	(0.000)	(0.000)
cons	−1.558***	−1.708***	−1.460***	−1.574***	−0.796	7.444***	2.889***	6.038***
	(0.000)	(0.000)	(0.000)	(0.000)	(0.433)	(0.000)	(0.000)	(0.001)
controls	no	yes	no	yes	no	yes	no	yes
*n*	933	933	933	933	933	933	933	933

Columns 7 and 8 show that although subjects who are mothers and subjects who are not mothers allocated more to world’s mothers than to world’s women, the difference between what was allocated to world’s mothers and world’s women is still significantly larger for subjects who are mothers themselves.

## 4. Study 2: design and hypotheses

We conducted our second study on Prolific where we ordered a sample of 400 subjects. We had a final sample of 319 subjects who passed attention and comprehension checks and did not report technical problems. All subjects were American citizens. The mean age was 39; 56% of the subjects had a university degree and 56% were male; 45% supported the Democratic party and 15% supported the Republican party. We paid £1.85 for subjects in the second study resulting in an average hourly rate of £8.53, which corresponded to $10.56 on the day of data collection (1 February 2023).

The study had two between-subjects conditions: a video condition and a control condition. The procedure of the study was as follows: first, we elicited demographic information. In the video condition, we showed subjects a video about globally shared biology before we asked comprehension check questions and measured opinions about the video and outcomes. In the control condition, we measured outcomes first before we showed the video, asked comprehension check questions and measured opinions about the video. This approach allowed us to screen out subjects who failed to answer the comprehension check questions correctly or reported technical problems in both conditions in order to avoid selective attrition.

The video featured a TED talk in which the journalist A. J. Jacobs demonstrates that all humans are biologically related to each other, share common ancestors and thus all belong to a great human family. He illustrates his point by showing that he is a distant cousin of various celebrities. He speaks about the concept of a global family tree encompassing all human beings and invites everybody to a global family reunion event in New York. Jacobs states the wish that humans should engage in less hostile and more friendly interactions knowing that we are all family.^6^ After the subjects had watched the video, we elicited whether subjects faced technical problems and asked two comprehension check questions.

As outcome variables, we measured fusion outcomes and monetary allocation outcomes. We used the same measurement tools as in the first study. We measured fusion with six groups: (i) humanity as a whole, (ii) all Americans, (iii) the subject’s extended family, (iv) the subject’s immediate family, (v) supporters of the Democrats, and (vi) supporters of the Republicans. Moreover, we measured behaviour in three money allocation decisions where subjects had to split $100 between two persons in each allocation decision. Subjects had to make allocation decisions between the following people: (1) a randomly selected person from anywhere in the world versus a randomly selected American, (2) a member of the extended family versus a randomly selected American, and (3) a member of the Democrats versus a member of the Republicans. As explained in study 1, the money allocation tasks are validated, i.e. it has been shown that hypothetical allocation decisions predict incentivized allocation decisions very accurately. In the following, we refer to ingroup bias, if a subject favoured the American in decision (1), favoured the member of the extended family in decision (2) and favoured one side over the other in decision (3). In the case of (2) and (3), both persons were Americans, but differed in other group affiliations, i.e. family member versus non-family member in (2) and political affiliation in (3). Therefore, we speak of ingroup bias even if both persons were Americans in (2) and (3).

We pre-registered three hypotheses:^7^ ^,^ ^8^

– **hypothesis 1 (H1):** state fusion with humanity as a whole will be larger in the treatment condition;
– **hypothesis 2 (H2):** the difference between state fusion with supporters of the Democrats and state fusion with supporters of the Republicans will be smaller in the treatment condition; and
– **hypothesis 3 (H3):** exposure to the video will reduce ingroup bias in all three hypothetical money allocations.

State fusion is a measure of fusion with a group at a given time and differs from the standard version only by adding the phrase ‘right now’ when asking participants which pictorial diagram best describes their relationship with the respective group. In the following, we just use the term ‘fusion’. We focus on state fusion to assess the immediate impact of the video. We cannot answer the question of how strongly the effects of interventions highlighting globally shared biology persist over time and leave that for future research.

While we are primarily interested in the effect of the treatment on fusion with humanity, we also tested whether appeals to globally shared biological essence have unifying potential within countries in H2. We pre-registered that we will use OLS regressions to analyse whether exposure to the video affects the relevant outcomes. We have not pre-registered and we did not perform multiple comparison corrections.

## 5. Study 2: results

**Figure 2.**
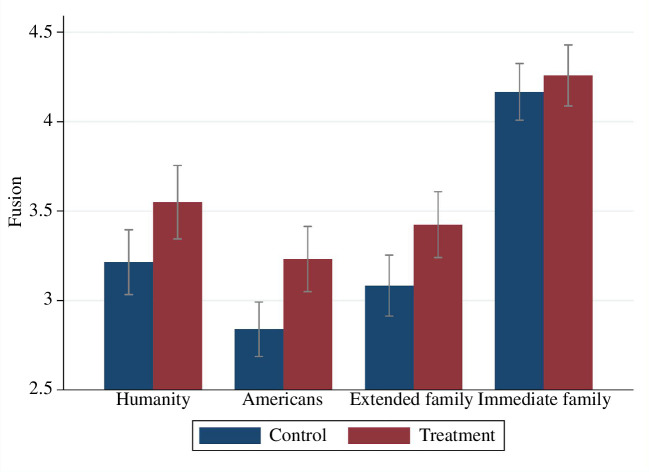
Fusion levels by treatment status. Confidence intervals have a confidence level of 0.95.

Fusion levels with humanity, Americans, extended family and immediate family by treatment status are presented in figure 2.

Exposure to the video increased fusion with humanity (OLS with robust s.e.; *b* = 0.335; *p* = 0.017; electronic supplementary material, table S21; H1), Americans (OLS with robust s.e.; *b* = 0.393; *p* = 0.001; electronic supplementary material, table S21) and the extended family (OLS with robust s.e.; *b* = 0.341; *p* = 0.008; electronic supplementary material, table S21). There was no significant effect on fusion with the immediate family (OLS with robust s.e.; *b* = 0.092; *p* = 0.441; electronic supplementary material, table S21).^9^ Shared ancestors and biology with the immediate family are perhaps already salient which might explain why exposure to the video did not generate a significant effect. Moreover, shared ancestors in the case of the immediate family are probably well-known to most members of the immediate family, whereas in the case of the extended family, shared ancestors might be less well-known personally and less salient, thus leaving some room for the treatment to increase their salience.

Exposure to the video decreased the absolute difference between fusion with supporters of the Democrats and fusion with supporters of the Republicans significantly (OLS with robust s.e.; *b* = −0.269; *p* = 0.030; electronic supplementary material, table S23). The video increased fusion with supporters of the outgroup party for subjects who support the Democrats (OLS with robust s.e.; *b* = 0.697; *p* < 0.001; electronic supplementary material, table S23) and for subjects who support the Republicans (OLS with robust s.e.; *b* = 0.772; *p* = 0.017; electronic supplementary material, table S23).^10^ There were no significant effects on fusion with supporters of the ingroup party for subjects who support the Democrats (OLS with robust s.e.; *b* = 0.291; *p* = 0.086; electronic supplementary material, table S23) and for subjects who support the Republicans (OLS with robust s.e.; *b* = 0.434; *p* = 0.121; electronic supplementary material, table S23).

The previous results suggest that notions of shared biology might not only increase fusion with humanity at large on a global level but also with the political opponent on a national level. Thus, notions of shared biology might not only help to address global problems but also help to mitigate conflicts along political lines on the national level. Moreover, the increases in fusion with humanity at large and the political opponent do not go hand in hand with decreases in fusion with the country or the political ingroup. Thus, high levels of fusion with different groups might coexist and fusion with different groups of people does not follow a zero-sum logic.

In the first money allocation decision, subjects had to split $100 between a random person who lives anywhere in the world and a random person who lives in the US. There was no significant treatment effect on allocations (OLS with robust s.e.; *b* = −$0.80; *p* = 0.735; electronic supplementary material, table S27). However, subjects allocated $51 to the American and $49 to the world citizen in the control condition, which does not significantly differ from the 50/50 split. Thus, ingroup bias did not exist and hence there was no room for the treatment to reduce it.

In the second allocation decision, subjects had to split $100 between a member of the subject’s extended family and a random person who lives in the US. We use the term ingroup bias if a subject allocated more to her extended family member than to the random person who lives in the US. Both persons might be Americans but differ in their family affiliation, thus one person belongs to the extended family ingroup and the other one does not. The effect on ingroup bias was not significant (OLS with robust s.e.; *b* = −$3.46; *p* = 0.234; electronic supplementary material, table S27).

Note, however, that the first and the second allocation decision were a measure of how much subjects cared about the respective person *relative* to the other one. Since the video increased fusion with humanity, Americans and the extended family, it is likely that treated subjects cared more strongly about both persons in both allocation decisions. In order to present evidence in support of this interpretation, we conducted further analyses that were not pre-registered. Table 3 presents evidence that fusion mattered for money allocation behaviour. Column 1 shows that in the first allocation decision, fusion with humanity significantly increased money allocation to the world citizen by $4.06 per point on the fusion scale if we control for fusion with Americans (OLS with robust s.e.; *p* = 0.001). This result shows that stronger fusion with humanity corresponds to higher shares allocated to the world citizen if we just compare subjects with the same fusion with Americans. Since fusion with Americans and fusion with humanity are positively and significantly correlated (*p* < 0.001), it makes sense to control for fusion with Americans to shed light on the relationship between fusion with humanity and the amount that is allocated to the world citizen.

**Table 3.** Effects of fusion on allocations 1 and 2. (*p < 0*.*1; **p < 0*.*05; ***p < 0*.*01. *p*−values in parentheses. *Notes:* Column 1 analyses the first allocation and column 2 analyses the second allocation decision. The two independent variables in both columns capture fusion with both sides of the respective allocation).

	(1)	(2)
	share American	share ext. family
fusion Americans	4.067***(0.006)	−4.256***(0.006)
fusion humanity	−4.065***(0.001)	
fusion ext. family		5.511***(0.001)
cons	52.43***(0.000)	66.61***(0.000)
*n*	319	319

Column 2 shows that in the second allocation, fusion with Americans significantly increased money allocation to Americans by $4.26 per point on the fusion scale if we control for fusion with the extended family (OLS with robust s.e.; *p* = 0.001). These results show that fusion matters for money allocation behaviour suggesting that higher fusion levels for both sides due to the treatment, balanced out which might explain the absence of significant effects in the univariate regressions.

In the third allocation decision, subjects had to split $100 between a member of the Democrats and a member of the Republicans. Subjects in the treatment group made allocations that were $6.05 closer to an equal split (OLS with robust s.e.; *p* = 0.016; electronic supplementary material, table S27), i.e. they showed less favouritism to one side or the other.^11^

## 6. Discussion

In study 1, we have shown that female subjects who are mothers were more fused with and allocated more money to other mothers than to women in general both in the national context and global context. These patterns did not emerge for female subjects who are not mothers. A difference in difference analysis revealed significant ‘shared motherhood’ bonuses on fusion and money allocations in both the national and global context that cannot be explained by age, education or party preferences. In study 2, we have provided causal evidence that notions of globally shared biology increase fusion with humanity, nation and extended family, and we have presented evidence that fusion influenced money allocation behaviour. Exposure to the treatment has also increased fusion with the political outgroup and decreased ingroup bias in money allocations between the political ingroup and outgroup.

A limitation of study 1 is that motherhood experiences—although shared across national, religious and ethnic boundaries—are not shared by everybody. Transformative experiences such as suffering, struggle or hope might perhaps be even more universally shared, but their global sharedness might also be more abstract than globally shared motherhood experiences. We leave it to future research to explore the potential of other globally shared experiences to foster bonding with humanity.

A limitation of study 2 is that while the video saliently highlights globally shared biology, it also includes other features. It references celebrities and politicians across the political spectrum including Barack Obama and George H. W. Bush. While these references might have effects on their own, politicians across the political spectrum are referenced which generates a certain political balance. Moreover, the video includes some humour and advocates directly for identification with humanity as a pro-social initiative. While these two factors might confound the effects of the pure informational aspects, they improve the validity of the design to capture the effects of normatively motivated attempts to foster bonding with humanity using a positive and light-hearted rhetorical style. However, it would be worthwhile for future research to test the effects of information provision about globally shared biology on bonding with humanity without the potential confounds discussed above, which might also facilitate to compare effects in multi-country studies.

Future research might test the effectiveness of globally shared transformative experiences and globally shared biology to foster bonding with humanity and prosocial action on a global scale in more applied settings and with additional outcomes like cooperation with foreigners, donations or support for policies that benefit humanity. Furthermore, future research might test the effectiveness of these two strategies in diverse populations. However, the existing literature on identity fusion presents clear evidence that the two pathways to fusion work cross-culturally, suggesting that globally shared transformative experiences and perceptions of globally shared biology are potentially effective strategies to foster fusion with and prosocial behaviour towards humanity in all human groups [34]. However, much more evidence on the effectiveness of these two strategies to foster bonding with humanity is needed. In the case of study 1, it remains an open question whether the same globally shared experiences are equally effective at fostering bonding with humanity across cultural contexts.^12^ In case of study 2, the talk we used as the treatment was given by a US citizen and was tailored towards a US audience. Therefore, future research might analyse whether a more culturally neutral intervention highlighting globally shared biology generates similar effects across countries and cultural contexts.^13^

These two strategies could transform efforts to address global collective action problems in at least three ways. First, systems of formal education, including national curricula, could incorporate a stronger emphasis on globally shared history and ancestry in ways that increase support for global cooperation in the next generation. Whereas it is already the norm in many countries to regard the teaching of national, religious and ethnic histories as a fundamental obligation of schools, it could (perhaps additionally) become a requirement to place more emphasis on global citizenship based on the shared collective experiences and common origins of humanity at large. Our second study showed that high levels of fusion with humanity and with other groups like nation or family are not mutually exclusive and can coexist. Thus, policies that strengthen fusion with humanity at large do not necessarily crowd out fusion with other relevant groups.

Second, political leaders with an interest in fostering cooperation on global challenges such as the climate crisis could use these findings to bring domestic audiences behind them and to galvanize coordinated action in international arenas.

Third, a wide range of transnational interest groups devoted to prosocial goals—ranging from non-governmental organizations tackling poverty or disaster management to religious organizations promoting peace and reconciliation—could more effectively harness the natural human propensity to bond and cooperate, by recognizing that much of what defines us as individuals is also fundamental to us as a global community. Although further work is needed to develop practical interventions capable of fulfilling this potential, the psychological foundations on which we must build are becoming increasingly evident.

## Data Availability

The datasets for study 1 and study 2 as well as the code (including all analyses) have been uploaded as part of the electronic supplementary material (all in Stata format) [37]. We used Stata 17.0.

## Footnotes

1 Differences between the pictorial scale used in [26] and the pictorial scale used in [27] are the relative sizes of the circles symbolizing the self and the group (the ‘self’ circle in [26] is smaller than the ‘group’ circle while they have equal sizes in [27]) and the degrees of overlap (ranging to full overlap in [26] and ranking to almost full overlap in [27]).

2 See AsPredicted no. 120175: https://aspredicted.org/HFS_FT4

3 We ran post hoc power analyses for our most relevant findings, yielding a power of 0.999 for H2a, H2b, H3a and H3b with an alpha of 0.05.

4 Mediation analyses with fusion as the mediator variable, motherhood status of the subject as the independent variable and money allocations as the outcome variable revealed significant indirect effects for the target groups US mothers and world’s mothers (see the electronic supplementary material, table S20).

5 All reported t-tests are two-tailed.

6 The url of the video is: https://www.youtube.com/watch?v=2_lBiFZ85d0

7 See Aspredicted no. 120278: https://aspredicted.org/HN5_2DD

8 A post hoc power analysis yielded a power of 0.779 for the results regarding H1, 0.706 for the results regarding H2, and 0.785 for the third money allocation with an alpha of 0.05 (the effects for the first two money allocations are not significant).

9 Results are robust with regards to the inclusion of control variables (age, gender, education, and party preferences) into the regressions (see the electronic supplementary material, tables S22, S24 and S28).

10 The number of Republicans in the main analysis was only 49. A post hoc power analysis yielded a power of 0.82 with an alpha of 0.05 for the treatment effect on fusion with Democrats for the subjects who support the Republicans. However, our primary interest lies in the overall treatment effect and not in comparing subjects who support the Democrats and subjects who support the Republicans.

11 A mediation analyses with the absolute difference between fusion with Democrats and fusion with Republicans as the mediator variable, treatment status as the independent variable and the absolute difference in money allocations as the outcome variable revealed a significant indirect effect (see the electronic supplementary material, table S31).

12 See Hanel *et al*. [35] on how similarities between diverse groups can improve attitudes towards an outgroup.

13 Kimel *et al*. [36] presented evidence that perceptions of genetic overlap between groups in conflict are related to peaceful behaviour towards the outgroup in a Middle East context.
